# Intra-observer and inter-observer errors in CT measurement of torsional profiles of lower limbs: a retrospective comparative study

**DOI:** 10.1186/s13018-015-0200-1

**Published:** 2015-05-15

**Authors:** Artemisia Panou, Dedorah Faith Stanitski, Carl Stanitski, Andrea Peccati, Nicola Marcello Portinaro

**Affiliations:** Department of Pediatric Orthopedics and Neuro-orthopedics, Humanitas Research Hospital, University of Milan, Via Manzoni 56, 20089 Rozzano, Milan Italy; Department of Translational Medicine, Orthopaedic and Traumatology Clinic, Humanitas Research Hospital, University of Milan, Rozzano, Milan Italy; Department of Orthopaedic Surgery, Medical University of South Carolina, Charleston, SC USA

**Keywords:** Torsional profiles, Intra-observer errors, CT measurements, Neck-shaft angle, Tibial torsion

## Abstract

**Background:**

The purpose of this study was to determine errors in measurement of torsional profiles (TP) (torsional femoral angle, torsional tibial angle, and femoral ankle angle) among four orthopedic surgeons, experts, and non-experts in measurement, and the learning curve.

**Methods:**

Twenty-six lower extremities of 13 patients with spastic diplegia candidates for femoral/tibial derotational osteotomy had preoperative bilateral computer tomography (CT) scan grams to establish the TP. Each measurement was done by four orthopedic surgeons, two experienced clinicians and interpreters of CT imaging and two with limited clinical and imaging assessment experiences. Images were blinded and the surgeons made three determinations at least 5 days apart; the three angles were measured each time for each limb. Intra-observer and inter-observer variability were determined using bias, standard deviation, and interclass correlation coefficient.

**Results:**

Significant inter-observer variability and bias were noted between experts and non-experts (average variability: ICC experts: 0.88 ± 0.15; ICC non-experts: 0.91 ± 0.09). For non-experts, excessive bias (25° and 14°) was observed. An associated improvement in bias with additional measurement experience indicated a potential significant learning curve for interpreting these studies. Less inter-observer variability was observed between experts.

**Conclusions:**

Measurement of TP is a reliable tool when used by experienced personnel, and their use as a preoperative tool should be reserved to ones with experience with such image assessments. Non-experts’ measurements produced a weak agreement when compared to experts’.

## Background

Lower extremity deformities in cerebral palsy (CP) include increased femoral anteversion and tibial medial or lateral torsion. The delay in normal physiologic resolution of torsion is primarily related to increased motor tone. Persistence of abnormal lower extremity bony torsion in combination with abnormal motor responses leads to dysfunction and clinical impairment. A femoral anteversion angle of >50° correlates well with the gross motor function classification system (GMFCS) score [1-5].

Standard clinical, routine radiographic assessments of lower extremity torsional abnormalities, ultrasound, or fluoroscopy may provide inadequate data to determine preoperative measurements in planned osteotomy correction cases [6-8]. Evaluations by computer tomography (CT) determinations for rotational deformities in the lower extremities have been recommended pre-surgery but the accuracy of such assessments has not been consistently documented [9].

To our knowledge, there are no previous reports comparing intra- and inter-observer agreement in measurement of torsional profiles (TP) with CT axial images with different levels of training in orthopedics. The purpose of this study was to assess the intra- and inter-observer agreement and accuracy, determine errors between measurements of torsional femoral angle (TFA), torsional tibial angle (TTA), and femoral ankle angle (FAA) of lower limbs provided by expert and non-expert orthopedic surgeons, and also determine the presence of a learning curve.

## Methods

Our study was performed according to the ethical standards of the Declaration of Helsinki (1964) and its later amendments. Acknowledgements by the Hospital’s Institutional Review Board were granted on February 2014, although using TP for pre-surgery decision in CP patients is routinely performed.

Inclusion criteria were diplegic patients who were candidates for derotational osteotomy of the lower limbs. All patients had CT scan grams using a multi-director row CT scanner (Somatomensaton Siemens, Germany) with 5.5 mm slice thickness. Patients were supine with the patella directly anterior with the hip and knee in as much extension as possible. No sedation was required for any case. Lower extremity restraints were used on the CT bed to maintain position throughout the study. A radiologist collected all the CT scan grams, concealed the patient’s information, and saved the images electronically. Each reviewer using a DICOM Viewer did measurements. Each CT scan was numbered and each observer measured the blinded images on three occasions with at least 5 days between readings. Lines for calculation were drawn on transparent paper during each measurement. The paper was eliminated after every measurement. Line placement was the choice of the individual assessor.

Each measurement was performed by four orthopedic surgeons, two experienced clinicians and interpreters of CT imaging (expert 1 and 2), and two orthopedic surgeons in training; one a first year resident and the other in his final year of the program (non-experts 1 and 2).

The following angles were measured:

- *TFA* is the angle formed between a line passing through the center of the femoral neck and a tangent line passing from the distal posterior femoral condyles and represents the angle of femoral version.
- *TTA* is the angle formed between a line tangent to the posterior surface of the proximal tibial plateau and a line passing through the mid-point of the tibial and fibular malleoli and represents the angle of tibial version.
- *FAA* is formed between a line passing through the center of the femoral neck and a line between the center of the tibial and fibular malleoli and represents the foot progression angle.

Intra-observer variability was done using the Bland-Altam method [10]. For each angle and for each observer, measurements were calculated by the difference from one measurement with respect to the previous measurement, e.g., measurement 1 vs 2, 3 vs 2, and 3 vs 1. Bias was determined for each measurement by taking the average of measured differences for each patient in addition to the standard deviation (SD). A bias of >10° was considered significant for each observer. For each measurement, the number of significant bias was calculated. For correlation of consecutive measurements carried out by the same observer, the interclass correlation coefficient (ICC) was used [11]. ICC is interpreted assessing the range between −1 and 1; agreement is stronger when ICC is equal to 1 or −1 and weak when equal to 0. Measurements were compared for each angle and for each observer for the presence of significant bias and the SD of the observations between measurements 3 vs 2; 3 vs 1 was used to identify presence of a learning curve in determining the data.

Inter-observer variability was assessed using the data generated by each observer and each angle for the first reading. All data combinations of the first reading for all observers yielded a total of six combinations for each of the six angles considered, e.g., 1 vs 2, 1 vs 3, 1 vs 4, 2 vs 3, 2 vs 4, and 3 vs 4, which were compared and an ICC determined for each combination.

## Results

From January 2013 to December 2013, 26 lower limbs of 13 patients with spastic diplegia scheduled for derotational osteotomy of the lower limbs were studied retrospectively. There were nine males and four females whose ages at the time of the study averaged 14.6 ± 6.6 years (range 8 to 35 years). Three patients, one male and two females were >16 years old at the time of the study (Table 1).

**Table 1:** **Population of the study**

	**Age < 16 years**	**Age ≥ 16 years**
Number of patients	10	3
Males	8	1
Females	2	2
Age (average ± SD)	12.30 ± 2.58	22.33 ± 10.97
Range	8–15	16–35

Intra-observer variability is shown in Table 2, and ICC is listed in the >10° column. For each observer and for each angle, two readings were taken into consideration: 1 and 2. ICC was calculated and results are reported in Table 3.

**Table 2:** **Intra-observer variability: for each expert/non-expert, two readings were taken into consideration (one and two) and SD and ICC were calculated**

	**Expert 1**			**Expert 2**			**Non-expert 1**			**Non-expert 2**		
	**Δ**_**m**_	**SD**	**>10**	**Δ**_**m**_	**SD**	**>10**	**Δ**_**m**_	**SD**	**>10**	**Δ**_**m**_	**SD**	**>10**
R-TFA(2–1)	0	2.31	0	0.69	1.55	0	1.15	3.34	0	2.25	3.49	0
R-TFA (3–1)	−0.31	2.43	0	0.92	1.50	0	1.92	3.55	0	3.08	5.30	1
R-TFA (3–2)	−0.31	1.97	0	0.23	2.09	0	0.77	2.42	0	0.83	2.33	0
R-TTA (2–1)	0.15	2.23	0	1.08	1.71	0	5.69	16.04	6	5	5.97	1
R-TTA (3–1)	−0.92	3.33	1	1.08	1.12	0	5.38	15.85	7	6.08	6.50	2
R-TTA (3–2)	−1.08	2.25	0	0.46	1.90	0	−0.31	2.10	0	1.08	3.89	0
L-TFA (2–1)	1.23	5.39	1	0.54	1.20	0	−0.77	3.14	0	1.33	4.19	0
L-TFA (3–1)	2.15	5.63	1	0.92	2.18	0	−1	3.29	0	5.33	5.00	3
L-TFA (3–2)	0.92	1.55	0	0.38	1.89	0	−0.23	1.48	0	4	3.74	1
L-TTA (2–1)	0.69	2.36	0	1.69	2.25	0	−0.92	8.76	2	3.33	4.64	1
L-TTA (3–1)	0.54	3.80	0	1.08	1.04	0	−1.15	9.01	3	4.33	5.77	2
L-TTA (3–2)	−0.15	3.21	0	−0.62	1.76	0	−0.23	3.22	0	1	4.20	0
R-FAA (2–1)	1.38	5.12	2	0.69	1.89	0	−1.69	4.80	0	1.17	4.63	0
R-FAA (3–1)	1.69	4.61	2	0.38	2.14	0	−1.54	4.88	1	1.17	4.45	0
R-FAA (3–2)	0.31	1.97	0	−0.31	1.55	0	0.15	2.82	0	0	4.92	1
L-FAA (2–1)	1.69	3.15	0	0.69	1.65	0	−2.54	7.32	1	2.42	3.82	0
L-FAA (3–1)	1.69	3.63	1	1.77	2.71	0	−3.69	7.18	3	4.25	6.22	2
L-FAA (3–2)	0	1.63	0	1.08	1.71	0	−1.15	4.49	1	1.83	3.59	0
Average	0.54	3.14		0.71	1.77		−0.01	5.76		2.69	4.59	
Total			8			0			25			14

**Table 3:** **ICC values to determine the inter-observer variability**

	**Expert 2**						**Non-expert 1**						**Non-expert 2**					
	**R-TFA**	**L-TFA**	**R-TTA**	**L-TTA**	**R-FAA**	**L-FAA**	**R-TFA**	**L-TFA**	**R-TTA**	**L-TTA**	**R-FAA**	**L-FAA**	**R-TFA**	**L-TFA**	**R-TTA**	**L-TTA**	**R-FAA**	**L-FAA**
Expert 1	0.98	0.97	0.99	0.99	0.93	0.98	0.80	0.60	0.70	–	–	–	–	0.79	0.72	0.72	0.56	–
Expert 2							0.92	0.15	0.58	–	0.81	0.71	0.02	0.81	0.87	0.70	–	–
Non-expert 1													0.78	0.97	0.95	–	0.70	–

The average values of ICC were:

- Intra-observer variability expert 1: ICC = 0.79 ± 0.16
- Intra-observer variability expert 2: ICC = 0.97 ± 0.02
- Average variability of experts: ICC = 0.88 ± 0.15
- Intra-observer variability non-expert 1: ICC = 0.97 ± 0.03
- Intra-observer variability non-expert 2: ICC = 0.87 ± 0.08
- Average variability of non-experts: ICC = 0.91 ± 0.09

The learning curve data showed significant statistical status for bias. SD and SD averages are reported in Table 4. Starting from these data we calculated:

**Table 4:** **Values of bias, SD, and average of the SD for each angle and for each observer (experts and non-experts)**

	**Bias 2–1**	**Bias 3–2**	**SD 2–1**	**SD 3–2**	**Δ Bias**	**Δ SD**	**Bias 2–1**	**Bias 3–2**	**SD 2–1**	**SD 3–2**	**Δ Bias**	**Δ SD**
	Expert 1						Expert 2					
R-TFA	1	0	2.31	1.97	0	−0.34	0	0	1.55	2.09	0	0.54
R-TTA	1	0	5.94	2.25	−1	−3.69	0	0	1.71	1.90	0	0.19
L-TFA	0	0	5.39	1.55	−1	−3.84	0	0	1.20	1.89	0	0.69
L-TTA	2	0	2.36	3.21	0	0.85	0	0	2.25	1.76	0	−0.49
R-FAA	0	0	5.12	1.97	−2	−3.15	0	0	1.89	1.55	0	−0.34
L-FAA	0	0	3.15	1.63	0	−1.52	0	0	1.65	1.71	0	0.06
Average			4.05	2.10	−0.67	−1.95			1.71	1.82	0.00	0.11
	Non-expert 1						Non-expert 2					
R-TFA	0	0	3.34	2.42	0	−0.92	0	0	3.49	2.33	0	−1.16
R-TTA	6	0	16.04	2.10	−6	−13.94	1	0	5.97	3.90	−1	−2.07
L-TFA	0	0	3.14	1.48	0	−1.66	0	1	4.19	3.74	1	−0.45
L-TTA	2	0	8.76	3.22	−2	−5.54	1	0	4.64	4.20	−1	−0.44
R-FAA	0	0	4.80	2.82	0	−1.98	0	1	4.63	4.92	1	0.29
L-FAA	1	1	7.32	4.49	0	−2.83	0	0	3.82	3.59	0	−0.23
Average			7.23	2.76	−1.33	−4.48			4.46	3.78	0.00	−0.68

The average ICC for each observer:

- Expert vs expert: ICC = 0.97 ± 0.02
- Expert vs non-expert: ICC = 0.65 ± 0.24
- Non-expert vs non-expert: ICC = 0.85 ± 0.13

The average ICC for each angle:

- R-TFA: 0.70 ± 0.39
- L-TFA: 0.72 ± 0.31
- R-TTA: 0.80 ± 0.13
- L-TTA: 0.80 ± 0.16
- R-FAA: 0.75 ± 0.28
- L-FAA: 0.85 ± 0.19

The data demonstrate the non-expert excessive bias (25° and 14°). These observers also gained the most improvement in bias with additional measurement experience. Experts showed less inter-observer variability.

## Discussion

Increased femoral anteversion and increased medial and tibial torsion are frequent abnormalities of the lower limbs in patients with CP. Persistence of lower extremity malalignment including torsional deformities leads to dysfunction and impairment of locomotion. Indications for surgical treatment are controversial.

CT is an accurate modality for rotational measurements and is frequently used to measure TP when difficulties arise to clinically assess such measurements [6]. Accurate measurements of the TP are fundamental to surgical correction planning.

In the literature, there are few works regarding accuracy and reproducibility of CT for the measurement of femoral and tibial torsion. Questions may arise on whether these CT assessments are reliable in the clinical setting and how much training is needed to provide accuracy of these critical measurements.

In our study, the first readings were used as reference measurements instead of an average of three assessments. A single individual in clinical practice to determine torsional deformities did this to mimic the single measurement.

A relatively high value (>10°) was chosen to determine when bias became positive. For example, a difference of >10° from normal values would lead to an indication for surgery if this value is used as a cutoff point. Regarding the implications for patients care, the presence of significant bias between two measurements could represent one situation where there was a surgical indication and the other, an indication for no surgery. Intra-observer variability had significant bias when experts and non-experts were compared. Expert number 1 had 25° of bias and non-expert number 2 had 14° of bias. The SD between experts (3.14 vs 1.77) is lower than that seen between non-experts (5.76 and 4.59).

In assessing the learning curve, we saw an improvement in measurement accuracy especially when non-experts were compared with each other. Non-expert number 1, the resident with no prior experience with the method, had the maximum number of significant bias results, but, also, had the greatest improvement in his data accuracy with progressive measurement experience. Non-expert number 2 showed no diminution of bias data with values increased and decreased on various readings. The improvement seen in the more senior resident values may be due to his observing others carrying out such measurements but had never performed them directly. The younger resident had never seen nor performed these measurements.

Relative to inter-observer variability, there was greater agreement between the two non-experts in respect to expert-non-expert comparisons. This was due to the non-experts providing inaccurate measurements in contrast to fewer measurement errors by the experts.

Our preliminary data suggest that TP measurements by CT provide a reliable tool when used by experienced personnel. The number of measurements and experience with CT TP determinations was not defined by our study. Additional study is required to differentiate when a transition from non-expert to expert occurs. In the interim, the ones with experience with such image assessments should use TP values of CT images as preoperative measures. Additional study must be done to develop the most accurate and reproducible method of determining femoral and tibial version, to compare measurements of radiologists and orthopedic surgeons, experts and non-experts, and highlight their differences.

## Conclusion

Measurement of TP is a reliable tool when used by experienced personnel and their use as a preoperative tool should be reserved to ones with experience with such image assessments. Non-experts’ measurements produced a weak agreement when compared to experts’.

## Abbreviations

CP: cerebral palsy
FAA: femoral ankle angle
GMFCS: gross motor function classification system
ICC: interclass correlation coefficient
SD: standard deviation
TFA: torsional femoral angle
TP: torsional profiles
TTA: torsional tibial angle
